# Hollow TiO_2_ Nanoparticles Capped with Polarizability-Tunable Conducting Polymers for Improved Electrorheological Activity

**DOI:** 10.3390/nano12193521

**Published:** 2022-10-08

**Authors:** Seungae Lee, Jungchul Noh, Suk Jekal, Jiwon Kim, Won-Chun Oh, Hyung-Sub Sim, Hyoung-Jin Choi, Hyeonseok Yi, Chang-Min Yoon

**Affiliations:** 1 Department of Chemical Engineering, Konkuk University, Seoul 05029, Korea; 2 McKetta Department of Chemical Engineering and Texas Material Institute, The University of Texas at Austin, Austin, TX 78712, USA; 3 Department of Chemical and Biological Engineering, Hanbat National University, Daejeon 34158, Korea; 4 Department of Advanced Materials Science and Engineering, Hanseo University, Seosan-si 31962, Korea; 5 Department of Aerospace Engineering, Sejong University, Seoul 05006, Korea; 6 Department of Polymer Science and Engineering, Inha University, Incheon 22212, Korea; 7 Program of Environmental and Polymer Engineering, Inha University, Incheon 22212, Korea; 8 Institute for Materials Chemistry and Engineering, Kyushu University, Fukuoka 816-8580, Japan

**Keywords:** electrorheological fluids, hollow TiO_2_ nanoparticles, surface modification, conducting polymers, space charge

## Abstract

Hollow TiO_2_ nanoparticles (HNPs) capped with conducting polymers, such as polythiophene (PT), polypyrrole (PPy), and polyaniline (PANI), have been studied to be used as polarizability-tunable electrorheological (ER) fluids. The hollow shape of TiO_2_ nanoparticles, achieved by the removal of the SiO_2_ template, offers colloidal dispersion stability in silicone oil owing to the high number density. Conducting polymer shells, introduced on the nanoparticle surface using vapor deposition polymerization method, improve the yield stress of the corresponding ER fluids in the order of PANI < PPy < PT. PT-HNPs exhibited the highest yield stress of ca. 94.2 Pa, which is 5.0-, 1.5-, and 9.6-times higher than that of PANI-, PPy-, and bare HNPs, respectively. The improved ER response upon tuning with polymer shells is attributed to the space charge contribution arising from the movement of the charge carriers trapped by the heterogeneous interface. The ER response of studied ER fluids is consistent with the corresponding polarizability results as indicated by the permittivity and electrophoretic mobility measurements. In conclusion, the synergistic effect of hollow nanostructures and conducting polymer capping effectively enhanced the ER performance.

## 1. Introduction

Electrorheological (ER) fluids are suspensions of electrically polarizable particles in insulating solvents [1,2,3]. These materials form viscoelastic solids with fibril-like structures under sufficiently strong applied electric fields [4,5,6,7]. The induced structural transformation can result in changes in the shear and tensile strengths, damping capacity, and internal friction [1,8]. These features are instantaneously reversible upon the removal of the field [9,10]. Such reversible tunability of viscoelastic properties can impart electric and hydraulic components with a low power consumption [11,12,13]. Therefore, electrofluidic systems can be used for a variety of applications, such as shock absorbers, dampers, transducers, and medical haptic devices [14,15,16].

Polarizability is the driving force for the observed ER response [17,18]. The dipole moments induced by the applied electric field cause surface charge distribution around particles [19,20]. The resulting interparticle interaction strengthens the fibrillated structure, and this effect is even more pronounced in materials with a high permittivity [21,22]. Various polarizable materials have been explored as ER fluids, such as conducting polymers, dielectric inorganics, and their composites [23,24,25,26,27]. Among these materials, TiO_2_ is considered a highly promising candidate for ER applications [28]. Although the effective permittivity of TiO_2_ dispersed in insulating solvents is smaller than that of its bulk counterpart, high potential energy can be stored even at a relatively weak electric field strength [29]. Additional dipole localization can be achieved by controlling the morphology, using fillers, doping, and performing surface modifications [10,19,30,31]. However, these techniques often result in colloidal instability, which is detrimental to the reversibility of the ER response [2,32]. Recent studies have illustrated that porous TiO_2_ nanoparticles (NPs) exhibit high dispersion stability while maintaining polarizability; however, a tunable polarizable structure with high dispersion stability has not yet been demonstrated [19,20].

A practical method to attain a tunable polarizable structure is through surface modification as interparticle interactions are strongly dependent on surface characteristics. Therefore, heterolayers comprising a series of thin layers of different materials are considered promising candidates to alter the polarizability and ER activity [33,34]. In this regard, the capping of particles with π-conjugated conducting polymers can be used to obtain space charge contribution to improve polarizability [35]. Intrinsically generated charge carriers in the π-conjugated systems are trapped in the interface, which hinders their movement [36]. Such space charge dynamics, therefore, offer an additional charge distribution mechanism [37]. For the space charge effect, the corresponding electrical conductivity should be approximately 10^−9^–10^−8^ S/m for achieving the ER response. A higher conductivity could lead to an electrical shortage in ER fluids [20,38]. Capping the particles with conducting polymers can also help to adjust the polarizability and conductivity by controlling the chain length of conjugated bonds through synthetic methods. We aim to develop polarizability-tunable nanostructures stabilized in an insulating medium toward high ER performance with reversible response.

Here, we report ER fluids of hollow TiO_2_ nanoparticles (HNPs) capped with different conducting polymers with polarization-tunable reversible responses. The HNPs were synthesized using SiO_2_ NPs as sacrificial templates by introducing a porous TiO_2_ layer as the shell in SiO_2_/TiO_2_ core/shell nanoparticles (CSNPs), followed by the etching of the silica core. The surfaces of the CSNPs and HNPs were capped with various conducting polymers, such as polythiophene (PT), polypyrrole (PPy), and polyaniline (PANI), using vapor deposition polymerization (VDP) method to further enhance the ER performance. The synthetic approach for polymeric shells leads to the tunable polarizability while retaining the dispersion stability in an insulating silicone oil, which has been a long-standing goal for ER applications. As a result, HNPs exhibit a higher ER response in contrast to CSNPs, owing to the increased dispersion stability and higher number density of ER fluids. Moreover, the ER performance increased in the order of PANI-, PPy-, and PT-HNPs. The ER response of the studied nanomaterials matches well with the corresponding polarizability strengths of the conducting polymers as proved by the permittivity and electrophoretic light scattering (ELS) measurements.

## 2. Materials and Methods

### 2.1. Materials

Tetraethyl orthosilicate (TEOS, 98.0%), titanium isopropoxide (TTIP, 97.0%), thiophene (99.0%), pyrrole (99.0%), aniline (99.5%), iron chloride (97%), and silicone oil (poly(methylphenylsiloxane), viscosity = 100 cSt) were purchased from Sigma-Aldrich Chemical Co. (Burlington, MA, USA). Ammonium hydroxide (28–30%), acetonitrile (99.8%), and absolute ethanol (EtOH, 99.5%) were purchased from Samchun Chemical Co. (Seoul, Korea). All chemicals were used without further purification.

### 2.2. Synthesis of SiO_2_/TiO_2_ CSNPs

SiO_2_/TiO_2_ CSNPs were synthesized using a typical sol-gel process. The SiO_2_ NPs were used as sacrificial core and prepared using the Stöber method. In a typical synthesis, TEOS (3.1 mL) was added to a solution of EtOH (98.0 mL), deionized water (2.0 mL), and ammonium hydroxide (5.0 mL) and stirred for 6 h at room temperature. Subsequently, the TiO_2_ layers were coated onto the SiO_2_ NPs. A mixture of TTIP (6 mL), absolute EtOH (36 mL), and acetonitrile (12 mL) was slowly added to the dispersed SiO_2_ NPs in EtOH at 4 °C. The reaction was performed for 12 h at 4 °C. The dispersion was then centrifuged at 8500 rpm for 25 min. The supernatant was discarded, and the precipitate was redispersed in EtOH (75.0 mL). The centrifugation-washing cycle was repeated three more times, and the precipitate was dried at room temperature overnight.

### 2.3. Fabrication of HNPs

HNPs were synthesized using the sonication-mediated etching and redeposition method. Dried CSNPs (0.4 g) were dispersed in deionized water (15 mL) via sonication, followed by stirring for 3 h. Ammonium hydroxide (3 mL) was added to the prepared dispersion, which was then sonicated for 12 h. The NPs were isolated via centrifugation at 10,000 rpm for 30 min. The supernatant was discarded, and the precipitate was redispersed in deionized water (20 mL). The centrifugation and washing steps were repeated three more times, and the final product was dried at room temperature overnight.

### 2.4. Surface Modification with Conducting Polymers

The surface of CSNPs and HNPs were capped with conducting polymers using a vapor deposition polymerization method. First, each CSNPs (0.3 g) and HNPs (0.3 g) were dispersed in deionized water (50.0 mL). Thereafter, FeCl_3_ (0.8 g) was added to both dispersions, and the mixture was stirred for 6 h. The FeCl_3_-soaked CSNPs and HNPs were collected via centrifugation at 8500 rpm for 25 min and dried at room temperature overnight. The NP surfaces were modified using a series of conducting polymers (PT, PPy, and PANI). CSNPs and HNPs were loaded into the VDP apparatus that was evacuated at 80 °C. Thereafter, 0.1 mL of the conducting polymer were carefully injected into a monomer chamber in the VDP apparatus to coat with the targeted conducting polymer. VDP was performed for 6 h. The final conducting polymer-capped CSNPs and HNPs were collected via centrifugation at 10,000 rpm for 30 min and dried overnight.

### 2.5. Characterization

Transmission electron microscopy (TEM) images of nanocrystals on continuous carbon-coated Cu grids were obtained using a Hitachi HF5000 system operated at 80.0 kV (Tokyo, Japan). NPs were drop-cast onto the TEM substrates from EtOH dispersions and dried overnight. Field-emission scanning electron microscopy (FE-SEM) images of the nanocrystals deposited on a Si wafer were obtained using a Hitachi S-4800 instrument operated at 10.0 kV with an in-lens detector (Tokyo, Japan). Fourier-transform infrared (FTIR) spectroscopy was performed using a Nicolet iS10 spectrometer (Thermo Fisher Scientific, Waltham, MA, USA). The NP powder was loaded onto a sample holder, and the spectra were acquired with 512 scans at a resolution of 4 cm^−1^. Dielectric properties were determined using an impedance analyzer (Solartron 1260, Bognor Regis, UK) equipped with a dielectric interface system (Solartron 1296). NPs dispersed in silicon oil (3.0 wt%) were loaded into the chamber. Permittivity measurements were performed by supplying an alternating current in the frequency range of 10^−^^2^ to 10^7^ Hz. The electrophoretic mobility of the materials was determined using an Otsuka ELS-8000 system (Tokyo, Japan). The electrical conductivity of the sample pellets was obtained using a Mitsubishi MCP-HT450 instrument (Tokyo, Japan) via a two-point method.

### 2.6. Investigation of ER Properties

The ER properties were studied using an MCR 302 rheometer (Anton Paar GmbH, Graz, Austria). ER fluids were prepared by dispersing the NPs in silicon oil. The concentrations of NPs in all ER fluids were 3.0 wt%, and no additives were added. Samples were loaded onto a cup (diameter = 30.0 mm and height = 30.0 mm) with a Couette geometry (diameter = 28.0 mm and height = 30.0 mm). The gap distance between the geometry and cup for the shear force was set to 1.0 mm on each side (total: 2.0 mm). The rotor was aligned to the center of the sample holder before measurements with automated setup. Mechanical shear (1.0 s^−^^1^) was applied for 5 min to obtain a stable response of the fluid. A high-voltage generator (HCN 7E-12500, FuG Elektronik GmbH, Schechen, Germany) was connected to the bottom of the cup and the top part of the rotor using alligator clips. The shear stresses were measured by varying the shear rate or by switching the electric field.

## 3. Results and Discussion

### 3.1. Fabrication of Various Conducting Polymer-Capped CSNPs and HNPs

**Figure 1:**
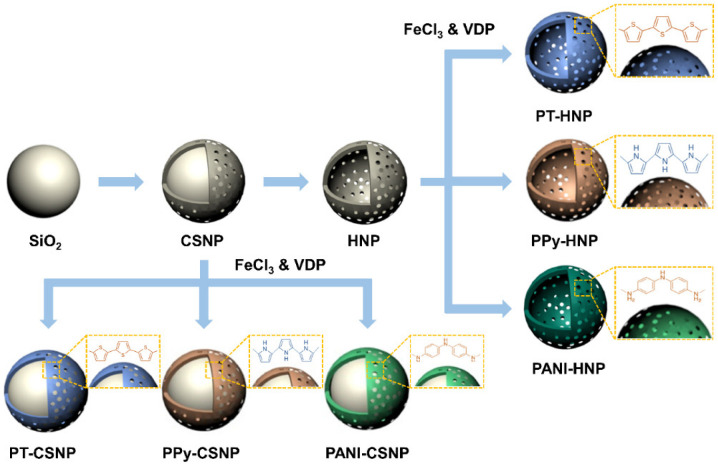
Schematic of the synthesis of SiO_2_/TiO_2_ core/shell nanoparticles (CSNPs) and hollow TiO_2_ nanoparticles (HNPs) capped with polythiophene (PT), polypyrrole (PPy), and polyaniline (PANI). CSNPs were synthesized using the sol-gel process. HNPs were prepared using the sonication-mediated etching and redeposition (SMER) method in a base solution containing ammonium hydroxide. Conducting polymer capping on CSNPs and HNPs was accomplished using the vapor deposition polymerization (VDP) method to obtain PT-, PPy-, and PANI-CSNPs and HNPs.

Figure 1 shows the schematic of the synthesis of CSNPs and HNPs capped with different conducting polymers, PT, PPy, and PANI. Spherical SiO_2_ NPs were synthesized as core templates using the Stöber method. The TTIP precursor in acetonitrile and EtOH was slowly added to the colloidal SiO_2_ solution that was cooled in an ice bath. Acetonitrile stabilizes TTIP and offers high oxidation tolerance in atmospheric air. The slow injection into the cooled solution resulted in TiO_2_ shell formation while suppressing the homogenous growth of TiO_2_ NPs. As the activation energy of heterogeneous nucleation is lower than that of the homogenous nucleation, maintaining mild reaction conditions is important for the formation of the core/shell structure. The porous structure is also essential for etching the SiO_2_ core by sonicating the NPs in an aqueous NH_4_OH solution. However, several SiO_2_ core fragments can be redeposited on the NP surface during sonication, resulting in the passivation of TiO_2_ shells by SiO_2_ fragments. This process is also referred to as the sonication-mediated etching and redeposition (SMER) method [20,31,39,40]. Both CSNPs and HNPs were then encapsulated by conducting polymers to modify their polarizability under electric fields. To obtain a thin-layer coating, the VDP method was employed. After soaking the NPs with FeCl_3_, the specific conducting polymer was added under a vacuum at 80 °C. This resulted in the formation of CSNPs and HNPs capped with PT, PPy, and PANI.

**Figure 2:**
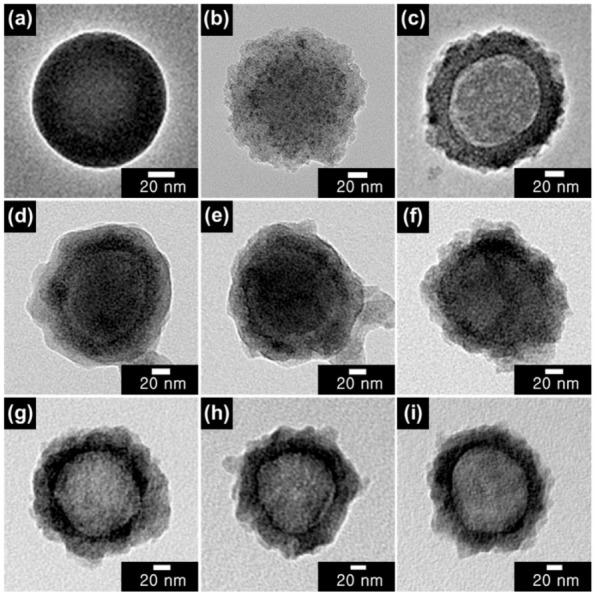
Transmission electron microscopy (TEM) images of (**a**) SiO_2_ nanoparticles (NPs), (**b**) CSNPs, (**c**) HNPs. Surfaces of CSNPs and HNPs were modified by different conducting polymers (PT, PPy, and PANI) using a vapor deposition polymerization (VDP) method. Images in (**d**–**f**) show PT-, PPy-, and PANI-CSNPs, respectively, while images in (**g**–**i**) show PT-, PPy-, and PANI-HNPs, respectively.

**Figure 3:**
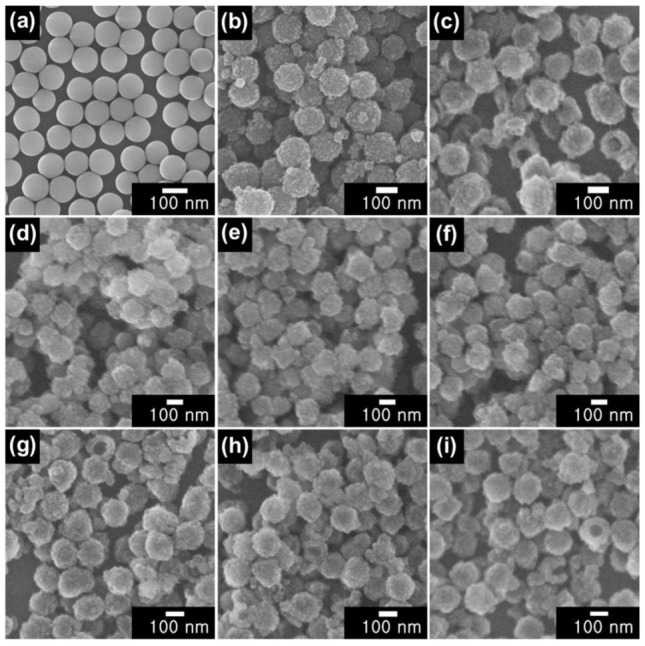
Scanning electron microscopy (SEM) images of (**a**) SiO_2_ NPs, (**b**) CSNPs, (**c**) HNPs. Images in (**d**–**f**) show PT-, PPy-, and PANI-CSNPs, respectively, while images in (**g**–**i**) show PT-, PPy-, and PANI-HNPs, respectively.

Figure 2 and Figure 3 show TEM and SEM micrographs of SiO_2_ NPs, CSNPs, and HNPs. Highly uniform spherical SiO_2_ NPs were synthesized with an average diameter of approximately 100 nm. Furthermore, the addition of TTIP to the colloidal SiO_2_ solution resulted in the formation of a porous TiO_2_ shell with a rough surface and an approximate thickness of 20 nm (Figure 2b). A few smaller particles observed in the SEM image are likely to be stand-alone TiO_2_ particles produced via homogeneous nucleation. However, the reaction mainly occurred on the SiO_2_ surface, indicating that mild reaction conditions favor the heterogeneous growth of the TiO_2_ shell, as expected. The removal of the SiO_2_ templates using the SMER method resulted in HNPs with a thickness of approximately 25 nm. The slight increase in thickness is attributed to the redeposition of SiO_2_ fragments on TiO_2_ shells. The contrast between TiO_2_ shells and the cavity is visible in the TEM image in Figure 2c. The pinhole structures observed in the SEM image in Figure 3c also confirm the hollow structure of the HNPs.

The capping of conducting polymers alters the morphology of CSNPs and HNPs, forming additional layers of PT, PPy, and PANI after the VDP method with thiophene, pyrrole, and aniline, respectively. Although the polymerization maintained the original core/shell and hollow structures, the resulting polymer shells on the as-synthesized NPs possessed a considerably more irregular shape. Owing to a weaker imaging contrast compared to that of SiO_2_ and TiO_2_ and because of the lower atomic number of constituent materials in polymers, the polymer coatings tend to appear brighter in TEM images (Figure 2). The SEM micrographs presented in Figure 3 clearly show that the pinhole structure in HNPs was also covered during the polymer capping process. Both TEM and SEM micrographs confirm the successful passivation of both CSNPs and HNPs using VDP method by capping the particles with different conducting polymers.

**Figure 4:**
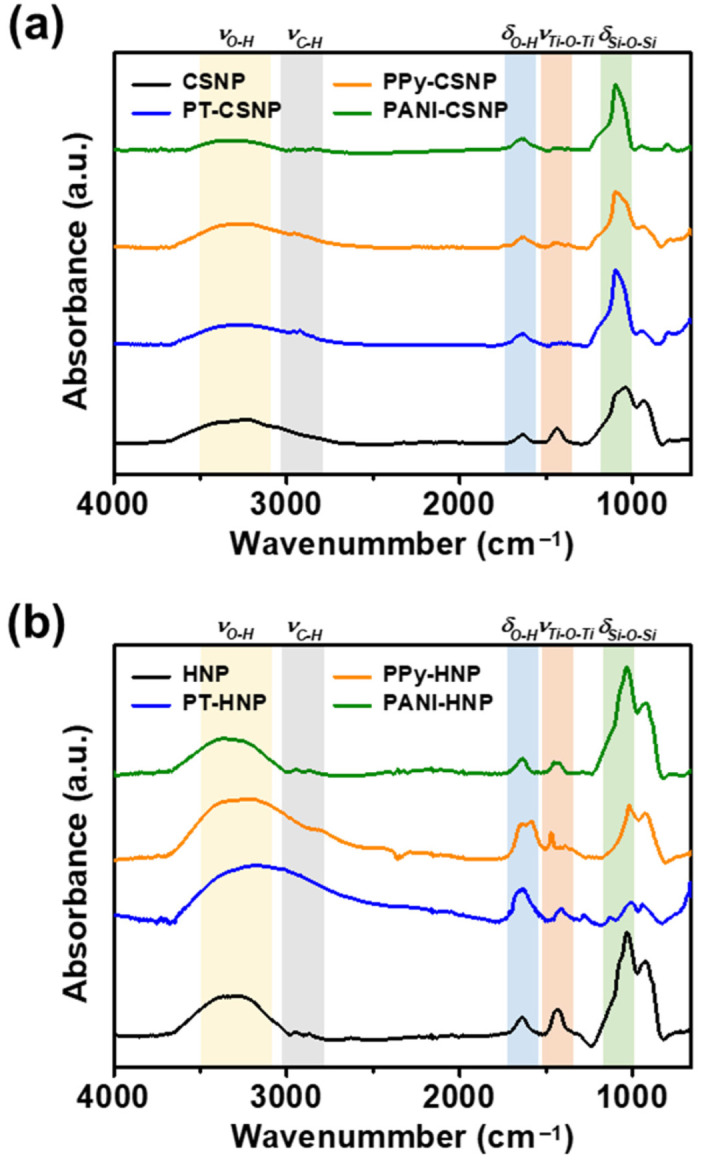
Representative Fourier-transform infrared (FTIR) spectra of (**a**) CSNPs and (**b**) HNPs measured before and after coating with PT, PPy, and PANI.

Figure 4 shows FTIR spectra of conducting polymer-capped CSNPs and HNPs. The representative spectrum of CSNPs shows stretching vibrations of Ti-O-Ti at 1437 cm^−1^ and bending vibrations of Si-O-Si at 1035 cm^−1^ [41,42]. The spectrum of HNPs shows red-shifted stretching vibrations of Ti-O-Ti at 1433 cm^−1^ and bending vibrations of Si-O-Si at 1028 cm^−1^. The shift of the absorbance band to lower energies could be attributed to the bonding deformation [43]. The presence of SiO_2_ features in the HNP spectrum supports the redeposition mechanism during the etching process. After capping with conducting polymers, additional peaks appeared in the range of 1800 to 1200 cm^−1^. However, the broad bending vibrations of the OH group and stretching vibrations of Ti-O-Ti overlap with some of the additional peaks. The overlap leads to a decrease in absorption intensity and red shift of the TiO_2_ peak, possibly due to the restricted stretching vibrations of TiO_2_ owing to the polymer shells. Additionally, all representative FTIR spectra show the stretching and bending vibrations of OH groups, indicating the passivation of NPs with the OH functional group or water moisture.

### 3.2. Investigation of ER Properties of Various Conducting Polymer-Capped CSNPs and HNPs

**Figure 5:**
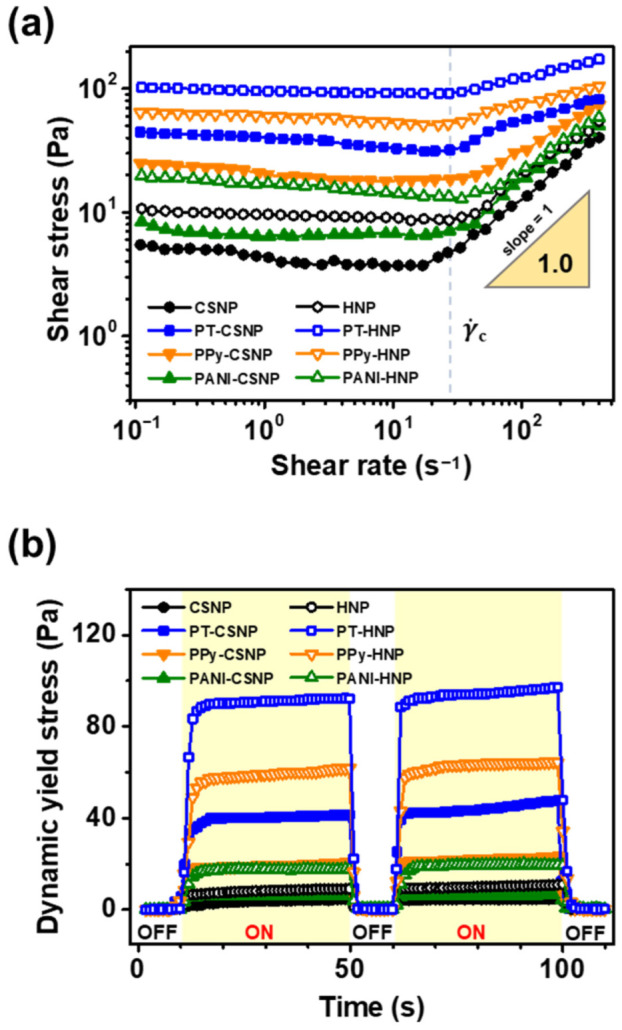
Electrorheological (ER) response of CSNPs and HNPs obtained before and after polymer capping with PT, PPy, and PANI. (**a**) Shear stress of NPs dispersed in silicone oil (3.0 wt%) measured by varying the shear rate at a constant electric field strength of 3.0 kV mm^−1^. (**b**) Dynamic yield stress of NPs in silicone oil dispersions (3.0 wt%) obtained by switching on and off the applied electric field of 3.0 kV mm^−1^ at a shear rate of 0.1 s^−1^.

Figure 5 shows the ER response of the conducting polymer-capped CSNPs and HNPs. Each NP sample was dispersed in silicone oil as an insulating solvent at a concentration of 3.0 wt%. Shear stresses of the ER fluids were obtained by varying the shear rate at an applied constant electric field strength of 3.0 kV mm^−1^. Under the applied electric field, induced charges are accumulated in the polarizable NPs, generating dipole moments that exert attractive forces among NPs that are aligned along the field. All ER fluids exhibit yielding behavior at relatively low shear rates. The appearance of a plateau region in Figure 5b indicates the formation of the fibril-like structure [44]. The electrostatic interparticle interaction is balanced with mechanical shear force; such yield stress appears until the shear rate reaches the critical value needed to break the NP arrays [45]. When the structure is no longer maintained at higher shear rates, the shear stress is linearly dependent on the mechanical shear rate. The shear-dependent yielding mechanism indicates the Bingham plastic behavior typical of ER fluids [46].

A comparison of the yield stresses is an indicator of the strength of attractive forces among polarized NPs. Higher yield stress should be observed for a more robust fibril-like structure. The yield stress of HNPs is ca. 9.8 Pa, which is 2.1-times higher than that of CSNPs. The observed increase in yield stress in HNPs can be attributed to the structural changes caused by the etching process. Since the intrinsic permittivity is higher for TiO_2_, the removal of SiO_2_ core increases in shear stress by forming robust fibril-like structure of assembled particles under an electric field. Moreover, the formation of the hollow structures reduced the mass of the material, resulting in the higher volume concentration compared to non-hollow structures at the same weight concentration when dispersed in silicone oil, which, in turn, leads to the formation of more compact fibril arrays under the applied electric field. A higher number density also increases the critical shear rate (${\dot{\gamma }}_{c}$), owing to which a linear slope appears in the shear stress curves. The critical shear rate is the minimum shear force required to break the yielding behavior. The formation of the compact arrays in HNPs under the applied electric field required a higher critical shear rate than that in CSNPs at a given weight fraction in ER fluids.

Improved shear stress ratios ($\tau_{\mathrm{HNP}}/\tau_{\mathrm{CSNP}}$) of HNPs to CSNPs for bare, PT-, PPy-, and PANI-capped samples were investigated to clarify the advantages in ER performance of HNPs (Figure S1). Regardless of capping of conducting polymers, the ratios are over 1, indicating the improvement of shear stress by etching process of the silica core. The effective permittivity could increase for HNPs because the dielectric parameters for TiO_2_ are higher than that of SiO_2_. The formation of hollow structures also reduces the particle density resulting in the increased number of particles in silicon oil at the same weight concentration. The compact fibril array under the electric field shows the improved shear stress. These factors result in the shear stress ratio of HNPs to CSNPs around 2 at a low shear rate of ~0.1 s^−1^. After the capping of conducting polymers, the ratios slightly increased with the following order: PPy > PANI > PT. However, it should be noted that the capping of PT could increase both CSNPs and HNPs significantly. Furthermore, the improvement ratios could be studied to understand the critical shear rate of HNPs and CSNPs, where the fibril-like structure starts to be destroyed by the minimum shear force. Physical meanings can be deduced from the increasing or decreasing onsets of shear stress ratio. Owing to greater number density in silicone oil, HNPs have higher critical shear rate than CSNPs, resulting in the increase in shear stress ratio at 0.1~100 s^−1^. The capping of PT exhibits the highest onset point, followed by PPy and PANI. The movement of charge carriers trapped by heterogenous interfaces, also known as space charges, can contribute to an enhanced interfacial polarization effect on ER response. The result indicates that the interfacial polarization occurs effectively in the following order: PT > PPy > PANI. Also, more carriers can be trapped on the interface having more defects. Since the etching process increases the porosity and defects, space charge contribution by conducting polymer shells occurs effectively for HNPs relative to CSNPs. In the high shear rate region, the fibril-like structures are broken for all samples, leading to the decrease in the shear stress ratio.

Shear stress and shear viscosity function of conducting polymers-capped CSNPs- and HNPs-based ER fluid as a function of shear stress without an electric field were investigated (Figure S2). Without the applied *E* field, all shear stress curves manifested Newtonian-like behavior with increasing shear rate, indicating the fluid-like characteristics. On the other hand, zero-field shear viscosities of all samples decreased with increasing shear rate, showing the Newtonian behavior. These zero-field experimental results of shear stresses and shear viscosities were ascribed to the dispersion characteristics of materials. As shown in Figure S2, all HNPs displayed higher than CSNPs for bare state and conducting polymers-coated state. With the etching of core material, dispersion stabilities of HNPs were enhanced compared with that of CSNPs. Furthermore, with the increased dispersion stabilities HNPs were able to disperse throughout the silicone oil, resulting in the higher viscosity of ER fluids than CSNPs-dispersed ER fluids without the application of *E* field. For the bare CSNP and HNP materials, zero-field shear stresses were higher compared to the conducting polymer-capped CSNPs and HNPs. Since conducting polymers adds weight to the bare CSNP and HNP, shear stresses of conducting polymer-capped CSNPs and HNPs were lower than bare materials. However, dielectric characteristics of conducting polymers enhances the ER performance of materials with the application *E* field.

Conducting polymer shells improve the yield stress by tuning the polarizability. Charge carriers were intrinsically generated by the π-conjugated systems of PT, PPy, and PANI. The movement of charge carriers trapped by heterogeneous interfaces, also known as space charges, can contribute to an enhanced polarization effect on the ER response [47]. In ER fluids, the addition of polymers enhanced the shear stress in the order of PANI < PPy < PT. The PT-HNPs exhibited the highest yield stress (ca. 94.2 Pa), which was 9.6-times higher than that of the as-synthesized HNPs. The detailed yield stress values for each ER fluid are listed in Table 1.

The dynamic yield stress indicates the reversibility of the ER response. The shear stress of each ER fluid was monitored by switching on and off the electric field of 3.0 kV mm^−1^ at a low shear rate of 0.1 s^−1^. All ER fluids showed a fast response to the applied electric field, reaching saturated shear stress within a few seconds. The magnitude of the shear stress under the applied field matches well with the yield stress obtained from the plateau region at the same electric field strength. The ER efficiency increased after the removal of SiO_2_ and followed the same trend: PANI < PPy < PT. The highest ER efficiency was obtained using PT-HNPs. Additionally, when the test was repeated, the response time and magnitude of the shear stress remained approximately the same. Therefore, repeated dynamic yield stress tests demonstrated the reversibility of the ER response.

### 3.3. Effect of Physical and Electrical Parameters on ER Response

**Figure 6:**
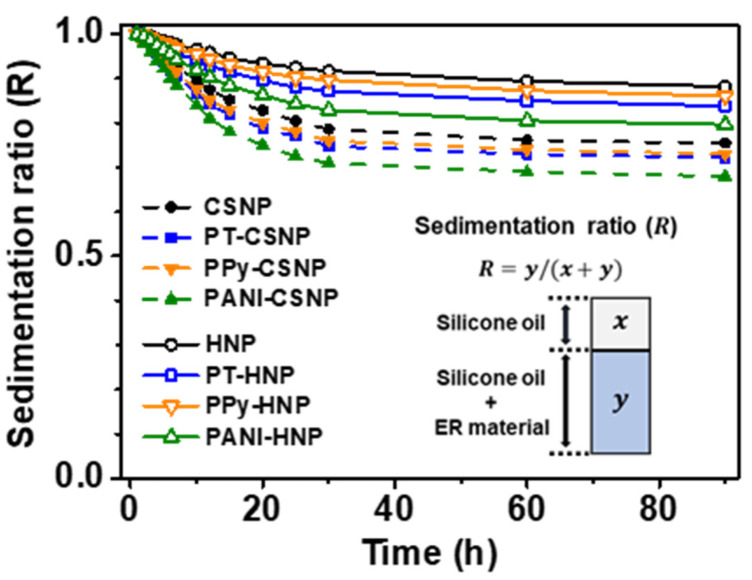
Colloidal dispersion stability of CSNPs and HNPs before and after the conducting polymer capping with PT, PPy, and PANI. Inset shows the sedimentation ratio of ER fluids.

The effect of various physical and electrical parameters on the ER response of conducting polymer-capped CSNPs and HNPs were investigated by measuring the dispersion stability, polarizability, electrophoretic mobility, and electrical conductivity. First, the colloidal dispersion stability of various conducting polymer-capped CSNP- and HNP-based ER fluids was examined (Figure 6). NPs (3.0 wt%) dispersed in silicone oil were placed in cylindrical vials with a diameter of 3 cm to observe sedimentation. Cloudy NP dispersions slowly settled and formed a transparent supernatant. The ratio of the height of the cloudy ER fluids over the clear silicone oil supernatant was defined as the sedimentation ratio (inset in Figure 6). The observation of the phase separation allows for the estimation of the colloidal stability. When the ratio remains high over time, it is visually apparent that particles are relatively well stabilized in the colloidal suspensions. In all ER fluids, the precipitation of NPs occurred within 30 h, followed by the saturation of the sedimentation ratio. Decreasing the density of the NPs resulted in a more stable colloidal dispersion. HNPs showed slower sedimentation than CSNPs owing to their hollow structure with increased dispersion stability. The dispersion stability slightly decreased upon the introduction of the conducting polymer shells. The saturated sedimentation ratio increased in the following order: PANI < PT < PPy.

**Figure 7:**
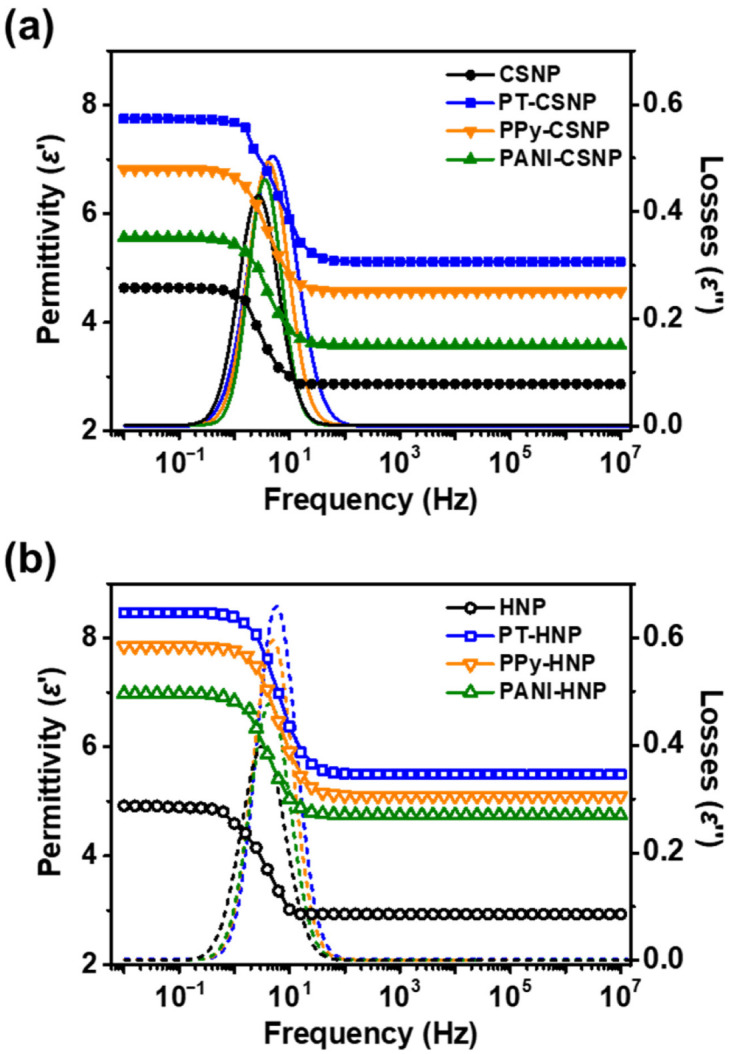
Permittivity and losses of (**a**) CSNPs and (**b**) HNPs dispersed in silicone oil (3.0 wt%) obtained by alternating the electric field with a strength of 3 kV mm^−1^ and with frequencies in the range of 10^−2^ to 10^7^ Hz.

The dielectric properties of ER materials are closely associated with ER performance since heterogeneous ER fluids can provide interfacial polarization (or Maxwell-Wagner-Sillars polarization) between the interface of ER materials and dispersing medium under an *E* field [48,49]. To investigate the dielectric characteristic and polarizability, frequency-dependent permittivity ($\varepsilon^{\prime }$) and losses ($\varepsilon^{\prime \prime }$) were determined for NPs in silicone oil dispersions (3.0 wt%) at an alternating electric field strength of 3.0 kV mm^−1^ (Figure 7). Specifically, polarization tendency (Δε) and rate of polarization (λ) of ER materials can be interpreted from the dielectric curves of $\varepsilon^{\prime }$ and $\varepsilon^{\prime \prime }$. For the former, Δε can be estimated by comparing the difference in the relative permittivity between the low and high frequency limits of each frequency. The static permittivity ($\varepsilon_{s}$) and permittivity at extremely high frequencies ($\varepsilon_{\infty }$) were obtained by linear extrapolation with frequency to 10^−2^ or 10^7^ Hz, respectively. For the latter, λ can be determined by the interpretation of $\varepsilon^{\prime \prime }$ curve associated with following equation

(1) $$\lambda =\frac{1}{2\pi f_{\max}}$$

where $f_{\max}$ is the frequency corresponding to the maximum/loss peak of $\varepsilon^{\prime \prime }$ curves [50,51]. The detailed dielectric parameters of ER fluids including Δε and λ are listed in Table 2.

In the perspective of ER activity, shear stress of ER fluids are generated by the formation of fibril-like structures under applied *E* field. With the application of *E* field, ER materials are subjected to generate interfacial polarization and fluid-like states are suddenly change to solid-like states. In this regard, polarization tendency and rate of polarization are key factors affecting the ER performance [52]. Many previous ER studies have reported the manipulation of structure and incorporation of materials with high polarizability [53]. Also, previous ER studies have reported that the large Δε and short λ values can provide benign effect on ER performance. It was clear that the HNPs-based ER fluids exhibited higher polarization tendency compared with that of CSNPs-based ER fluids. This phenomenon is attribute to the successful etching of SiO_2_ core from the CSNPs to attain HNP with higher contents of TiO_2_, which has naturally higher permittivity than SiO_2_ [54]. Also, internal hollow spaces of HNPs may provide additional charge accumulation sites [55]. Additionally, capping of conducting polymers further improved the polarizability, showing the same trend as in the ER response measurements (PANI < PPy < PT). The highest polarizability was observed for PT-HNPs, with Δε value of ca. 2.93. Similarly, λ of HNPs were shorter compare to the CSNPs indicating that the polarization rate of materials increased by acquiring the hollow structure, which synergistically attribute to the enhancement in permittivity and dispersion stability. With the short λ, HNPs are able to build up more numbers of fibril-like structures compared to CSNPs, resulting in higher the ER performance.

Electrophoretic light scattering (ELS) was performed on conducting polymer-capped NPs to gain more insights into the polarizability effect (Table 3). Similar to the ER response, the electrophoretic velocity ($v_{ep}$) increased with increasing NP polarizability. Notably, the phase shift of an incident oscillating electric field caused by the Doppler effect depends on electrophoretic mobility [56].

(2) $$\mu_{ep}=v_{ep}/E$$

The particle motion caused by an alternating electric field increased upon capping both CSNPs and HNPs with conducting polymer shells in the following order: PANI < PPy < PT, which is consistent with the permittivity and ER response results. PT-HNPs exhibited the highest electrophoretic mobility of ca. 3.6 × 10^−4^ cm^2^/V∙s. The results show that the electrophoretic particle motion increased with increasing polarizability.

Charge carriers in conducting polymers contribute to the observed increased polarizability [57]. NPs, which were originally insulating materials, became conductive after capping with PT, PPy, and PANI owing to the generation of delocalized charge carriers in the π-conjugated backbones. The films of NPs on Si wafers followed the same polarizability and ER trends (PANI < PPy < PT). PT-HNPs showed the highest conductivity of ca. 8.9 × 10^−9^ S/m. The other samples exhibited slightly reduced conductivities in the same order of magnitude. These values are lower than those of conducting polymers in solution. The possible explanation for these results is that the conducting polymers could have relatively short backbones with branched chains owing to the vapor-phase synthesis at a relatively high temperature of 80 °C. In addition, in heterostructures, charge carriers could be trapped in the interfaces, so that the space charge contributes to the polarizability without electrical shortage in ER applications.

## 4. Conclusions

In conclusion, HNPs capped with different conducting polymers were successfully synthesized with polarization-tunable ER response. The resulting HNPs dispersed in the silicone oil exhibited excellent yielding behavior under an external electric field. The structural transformation of CSNPs into HNPs increased the ER response by increasing the number density and dispersion stability. We also demonstrated that the ER performance can be further enhanced by capping the as-produced NPs with conducting polymer shells using a vapor deposition polymerization method. The ER response increased in the following order: PANI < PPy < PT. The PT-HNPs exhibited the highest yield stress of ca. 94.2 Pa, which was 5.0-, 1.5-, and 9.6-times higher than that of PANI-, PPy-, and bare HNPs, respectively. The ER results are consistent with the permittivity difference ($\Delta \varepsilon =\varepsilon_{S}-\varepsilon_{\infty }$), electrophoretic mobility, and electrical conductivity trends. We believe that the space charges originating from the movement of trapped charge carriers in conducting polymers lead to improved polarizability and corresponding ER response.

## Figures and Tables

**Table 1:** Yield stresses of various conducting polymer-capped CSNPs and HNPs dispersed in silicone oil (3.0 wt%) at a constant electric field strength of 3 kV mm^−1 *a*^.

Sample	Yield Stress (Pa)
SiO_2_/TiO_2_ CSNPs	4.6 ± 0.1
PT-CSNPs	42.2 ± 0.6
PPy-CSNPs	21.0 ± 0.5
PANI-CSNPs	6.3 ± 0.1
TiO_2_ HNPs	9.8 ± 0.4
PT-HNPs	94.2 ± 0.9
PPy-HNPs	62.6 ± 0.7
PANI-HNPs	19.0 ± 0.4

*^a^* A coverage factor (*k*) of 2 was used to calculate the values of expanded uncertainty.

**Table 2:** Dielectric parameters of CSNPs and HNPs capped with different conducting polymers and dispersed in silicone oil (3.0 wt%) *^a^*.

Sample	$\varepsilon_{s}$	$\varepsilon_{\infty }$	Δε b	${f_{\max}\;}^{c}$ (Hz)	${\lambda \;}^{d}$ (ms)
SiO_2_/TiO_2_ CSNPs	4.63 ± 0.05	2.85 ± 0.04	1.78 ± 0.04	2.44 ± 0.08	65.2 ± 2.2
PT-CSNPs	7.71 ± 0.07	5.14 ± 0.06	2.57 ± 0.06	4.36 ± 0.13	36.5 ± 1.1
PPy-CSNPs	6.78 ± 0.08	4.56 ± 0.06	2.22 ± 0.07	3.69 ± 0.10	43.1 ± 1.2
PANI-CSNPs	5.60 ± 0.06	3.56 ± 0.06	2.04 ± 0.06	3.19 ± 0.11	49.9 ± 1.7
TiO_2_ HNPs	4.88 ± 0.08	2.97 ± 0.06	1.91 ± 0.07	2.94 ± 0.07	54.1 ± 1.3
PT-HNPs	8.45 ± 0.09	5.52 ± 0.07	2.93 ± 0.08	5.15 ± 0.15	30.1 ± 0.9
PPy-HNPs	7.85 ± 0.09	5.07 ± 0.07	2.78 ± 0.08	4.45 ± 0.12	35.8 ± 1.1
PANI-HNPs	6.97 ± 0.08	4.74 ± 0.07	2.23 ± 0.07	3.93 ± 0.10	40.5 ± 1.0

*^a^* Dielectric properties were determined using an impedance analyzer (1260, Solartron) coupled with a dielectric interface (1296, Solartron). A coverage factor (*k*) of 2 was used to calculate the values of expanded uncertainty. *^b^* The difference between $\varepsilon_{s}$ and $\varepsilon_{\infty }$ ($\Delta \varepsilon =\varepsilon_{s}-\varepsilon_{\infty }$) is associated with the achievable polarizability. *^c^* The local frequency of the peak from the dielectric losses $\varepsilon^{\prime \prime }$ and the $f_{\max}$ values were measured by non-linear regression using OriginPro. *^d^* The relaxation time was measured using λ=1/($2\pi f_{\max}$) relation ($f_{\max}$ is the frequency corresponding to the maximum/loss peak of losses curve).

**Table 3:** Electrophoretic mobility and electric conductivity of CSNPs and HNPs before and after the conducting polymer capping with PT, PPy, and PANI *^a^*.

Sample	Electrophoretic Mobility *^b^* (cm^2^/V∙s)	Electrical Conductivity *^c^* (S/m)
PT-CSNPs	(3.2 ± 0.2) × 10^−4^	(7.6 ± 0.3) × 10^−9^
PPy-CSNPs	(2.7 ± 0.1) × 10^−4^	(5.5 ± 0.2) × 10^−9^
PANI-CSNPs	(2.5 ± 0.2) × 10^−4^	(2.2 ± 0.1) × 10^−9^
PT-HNPs	(3.6 ± 0.3) × 10^−4^	(8.9 ± 0.3) × 10^−9^
PPy-HNPs	(2.9 ± 0.2) × 10^−4^	(6.3 ± 0.3) × 10^−9^
PANI-HNPs	(2.6 ± 0.2) × 10^−4^	(3.4 ± 0.2) × 10^−9^

*^a^* A coverage factor (*k*) of 2 was used to calculate the values of expanded uncertainty. *^b^* Electrophoretic mobility values were acquired using an electrophoretic light scattering (ELS) apparatus (ELS-8000, Photal, Otsuka Electronics, Japan). *^c^* Electrical conductivity values were determined by the two-point method on the pellet-form sample.

## Data Availability

Data are contained within the article.

## Supplementary Materials

The following supporting information can be downloaded at: https://www.mdpi.com/article/10.3390/nano12193521/s1; Figure S1: Improvement shear stress ratio ($\tau_{HNP}/\tau_{CSNP}$) of bare, PT-, PPy-, and PANI-capped HNPs to CSNPs; Figure S2: Shear stress and viscosity of CSNPs and HNPs measured by varying the shear rate without an electric field.

## Abbreviations

CSNPs	SiO_2_/TiO_2_ Core/Shell Nanoparticles
ELS	Electrophoretic Light Scattering
ER	Electrorheology
FTIR	Fourier-Transform Infrared
HNPs	Hollow TiO_2_ Nanoparticles
NPs	Nanoparticles
PANI	Polyaniline
PANI-CSNPs	Polyaniline-capped SiO_2_/TiO_2_ Core/Shell Nanoparticles
PANI-HNPs	Polyaniline-capped Hollow TiO_2_ Nanoparticles
PPy	Polypyrrole
PPy-CSNPs	Polypyrrole-capped SiO_2_/TiO_2_ Core/Shell Nanoparticles
PPy-HNPs	Polypyrrole-capped Hollow TiO_2_ Nanoparticles
PT	Polythiophene
PT-CSNPs	Polythiophene-capped SiO_2_/TiO_2_ Core/shell Nanoparticles
PT-HNPs	Polythiophene-capped Hollow TiO_2_ Nanoparticles
SEM	Scanning Electron Microscopy
SMER	Sonication-Mediated Etching and Redeposition
TEM	Transmission Electron Microscopy
TEOS	Tetraethyl Orthosilicate
TTIP	Titanium Isopropoxide
VDP	Vapor Deposition Polymerization
