# Supplementary material for: Hollow TiO2 Nanoparticles Capped with Polarizability-Tunable Conducting Polymers for Improved Electrorheological Activity
Source: Nanomaterials (Basel). 2022 Oct 8;12(19):3521. doi: 10.3390/nano12193521 (PMC9565313; doi:10.3390/nano12193521)
Supplement: Supplementary file 1 [file nanomaterials-12-03521-s001.zip › nanomaterials-1905456-supplementary.pdf]

nanomaterials

Supplementary Materials

Hollow TiO_2 Nanoparticles Capped with Polarizability-Tunable Conducting Polymers for Improved Electrorheological Activity

Seungae Lee [1], Jungchul Noh [2], Suk Jekal [3], Jiwon Kim [3], Won-Chun Oh [4], Hyung-Sub Sim [5], Hyoung-Jin Choi [6,7], Hyeonseok Yi [8] and Chang-Min Yoon [3,*]

1 Department of Chemical Engineering, Konkuk University, Seoul 05029, Korea
2 McKetta Department of Chemical Engineering and Texas Material Institute, The University of Texas at Austin, Austin, TX 78712, USA
3 Department of Chemical and Biological Engineering, Hanbat National University, Daejeon 34158, Korea
4 Department of Advanced Materials Science and Engineering, Hanseo University, Seosan-si 31962, Korea
5 Department of Aerospace Engineering, Sejong University, Seoul 05006, Korea
6 Department of Polymer Science and Engineering, Inha University, Incheon 22212, Korea
7 Program of Environmental and Polymer Engineering, Inha University, Incheon 22212, Korea
8 Institute for Materials Chemistry and Engineering, Kyushu University, Fukuoka 816-8580, Japan
* Correspondence: cmyoon4321@hanbat.ac.kr; Tel.: +82-42-821-1528; Fax: +82-42-821-1593

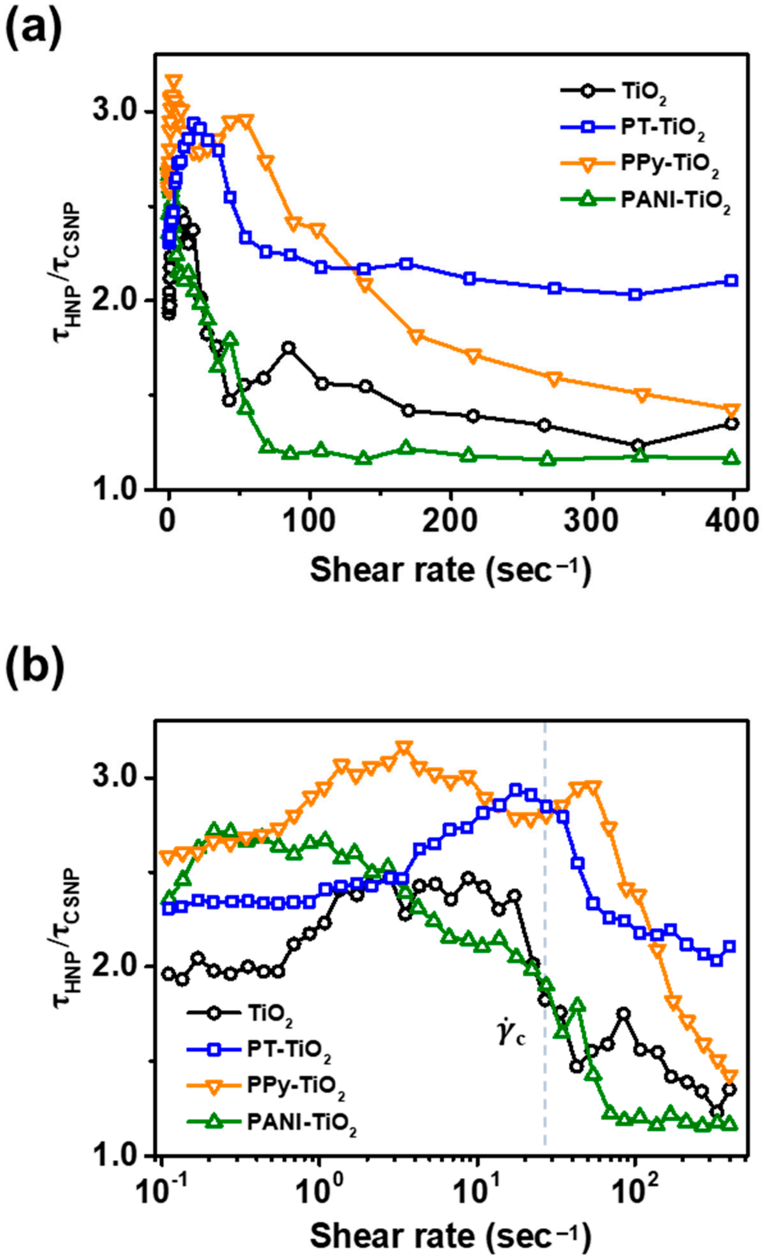

Figure S1. Improvement shear stress ratio (τ_{HNP}/τ_{CSNP}) of bare, PT-, PPy-, and PANI-capped HNPs to CSNPs measured by varying the shear rate (**a**) as a decimal scale and (**b**) a log scale at a constant electric field strength of 3.0 kV mm^{-1}.

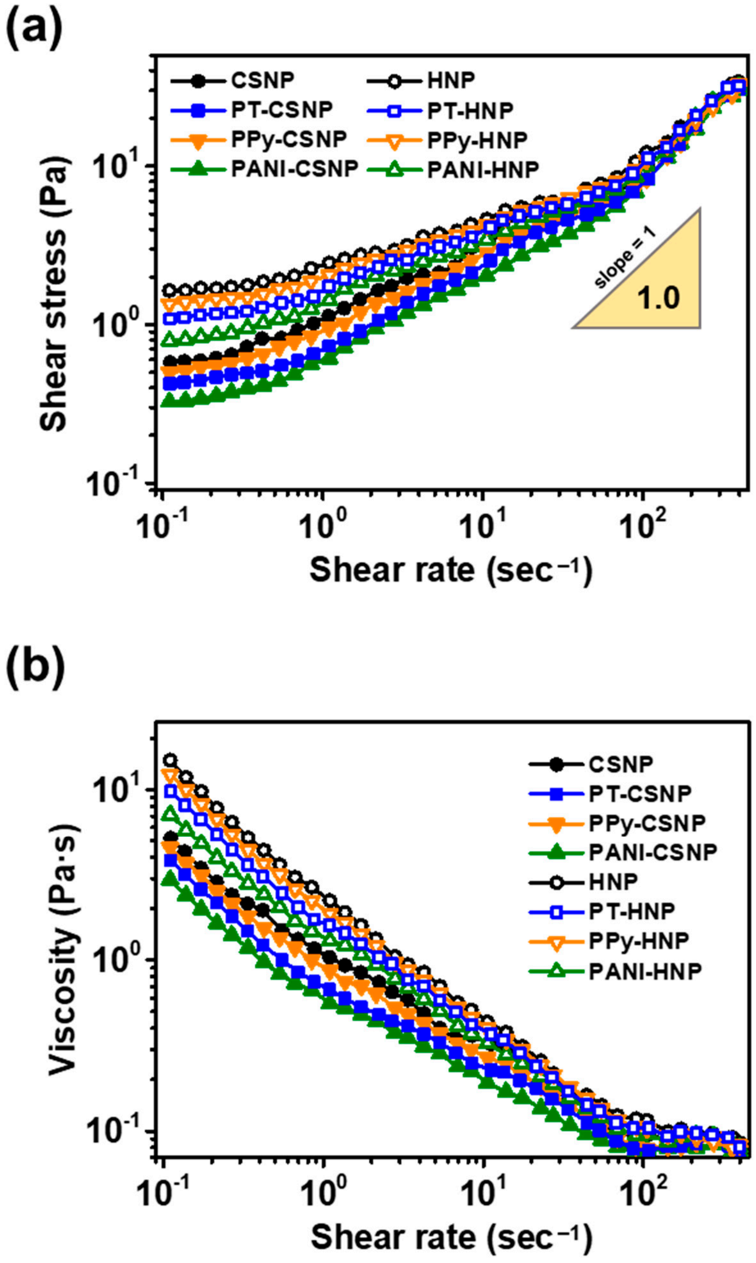

Figure S2. (**a**) Shear stress and (**b**) viscosity of CSNPs and HNPs obtained before and after polymer capping with PT, PPy, and PANI dispersed in silicone oil (3.0 wt%) measured by varying the shear rate without an electric field.
